# Fast functional imaging of multiple brain regions in intact zebrafish larvae using Selective Plane Illumination Microscopy

**DOI:** 10.3389/fncir.2013.00065

**Published:** 2013-04-09

**Authors:** Thomas Panier, Sebastián A. Romano, Raphaël Olive, Thomas Pietri, Germán Sumbre, Raphaël Candelier, Georges Debrégeas

**Affiliations:** ^1^CNRS/UPMC Laboratoire Jean Perrin, Université Paris 6Paris, France; ^2^Ecole Normale Supérieure, Institut de Biologie de l'ENS, IBENSParis, France; ^3^Inserm, U1024Paris, France; ^4^CNRS, UMR 8197Paris, France

**Keywords:** zebrafish model system, spontaneous activity, correlation analysis, neuroimaging, imaging, three-dimensional, light-sheet imaging

## Abstract

The optical transparency and the small dimensions of zebrafish at the larval stage make it a vertebrate model of choice for brain-wide *in-vivo* functional imaging. However, current point-scanning imaging techniques, such as two-photon or confocal microscopy, impose a strong limit on acquisition speed which in turn sets the number of neurons that can be simultaneously recorded. At 5 Hz, this number is of the order of one thousand, i.e., approximately 1–2% of the brain. Here we demonstrate that this limitation can be greatly overcome by using Selective-plane Illumination Microscopy (SPIM). Zebrafish larvae expressing the genetically encoded calcium indicator GCaMP3 were illuminated with a scanned laser sheet and imaged with a camera whose optical axis was oriented orthogonally to the illumination plane. This optical sectioning approach was shown to permit functional imaging of a very large fraction of the brain volume of 5–9-day-old larvae with single- or near single-cell resolution. The spontaneous activity of up to 5,000 neurons was recorded at 20 Hz for 20–60 min. By rapidly scanning the specimen in the axial direction, the activity of 25,000 individual neurons from 5 different z-planes (approximately 30% of the entire brain) could be simultaneously monitored at 4 Hz. Compared to point-scanning techniques, this imaging strategy thus yields a ≃20-fold increase in data throughput (number of recorded neurons times acquisition rate) without compromising the signal-to-noise ratio (SNR). The extended field of view offered by the SPIM method allowed us to directly identify large scale ensembles of neurons, spanning several brain regions, that displayed correlated activity and were thus likely to participate in common neural processes. The benefits and limitations of SPIM for functional imaging in zebrafish as well as future developments are briefly discussed.

## Introduction

Cognitive processes generally implicate extended neural networks spanning several areas of the brain. In order to shed light on the neural basis of these processes, it is thus necessary to simultaneously monitor the dynamics of multiple brain regions with single-cell resolution (Alivisatos et al., 2012). In striking contrast with this requirement, current experimental methods sample a small number of neurons located in a single brain area. Silicon-based nanoprobes allow electrophysiologists to simultaneously record up to a few hundred neurons (Du et al., 2011; Stevenson and Kording, 2011). Their future development is however limited by the intrinsically invasive nature of the technique. In the last two decades, functional imaging approaches, in which the neurons' spiking dynamics is monitored *via* the increase in fluorescence of calcium-binding reporters, have allowed to partially circumvent this limitation (Grienberger and Konnerth, 2012). Further assets of neuro-imaging, with respect to electrophysiology, include the accurate localization of the monitored neurons, the possibility to distinguish cell identity using specific markers and the precise manipulation of neural activity using optogenetic methods (Wyart et al., 2009).

Imaging of three-dimensional fluorescent tissues requires optical sectioning, which is generally obtained by using confocal or two-photon microscopy (Denk et al., 1990; Yuste and Denk, 1995; Bollmann and Engert, 2009). These point-scanning microscopy (PSM) approaches in turn impose a drastic limit in recording speed: in order to collect a significant number of photons in a given voxel, a minimum laser dwelling time of the order of 1 μs is needed (Holekamp et al., 2008). This yields a bound of roughly 10^6^ voxels per second in acquisition rate, which, even with optimized scanning trajectories (Salomé et al., 2006; Lillis et al., 2008; Grewe et al., 2010; Katona et al., 2012), limits the number of neurons that can be dynamically recorded.

In recent years, selective-plane illumination microscopy (SPIM) has been rediscovered in the context of embryo development (Mertz, 2011; Tomer et al., 2011; Weber and Huisken, 2011) and physiology (Huisken et al., 2004; Verveer et al., 2007; Keller and Dodt, 2012). In this imaging configuration, optical sectioning is performed through side-on illumination of the sample by a thin (micrometer-thick) laser sheet, whereas fluorescence photons are collected by a camera whose optical axis is orthogonal to the illumination plane. One of the significant assets of this approach, compared to PSM, lies in the fact that different regions in the focal plane are simultaneously illuminated and are thus exposed for a much longer time in average. As a result, the data throughput is in practice limited by the camera transfer rate, which is currently of the order of a few hundreds of Mpixels per second for highly sensitive sensors.

The strongest limitation of light-sheet microscopy compared to epifluoresence methods lies in the need to access the sample from two orthogonal axis. This is a severe constraint when dealing with large organs such as mammalian brain (Engelbrecht et al., 2010), which may explain why this method has not yet received a lot of attention from neurophysiologists. The only functional imaging experiments using SPIM that have been reported so far were performed on excised mice's vomeronasal organs (Holekamp et al., 2008; Turaga and Holy, 2012). Although the effective penetration depth was quite modest (of the order of 150 μm), this experiment did prove to produce simultaneous recordings of an unprecedented number of neurons with single-cell resolution.

Zebrafish is an ideal candidate for the use of SPIM-based *in vivo* functional imaging. At the larval stage, its brain is transparent and relatively small (typically 200 × 500 × 1000 μm) which makes it fully amenable for PSM-based calcium imaging (Higashijima et al., 2003; Niell and Smith, 2005; Ramdya and Engert, 2008; Sumbre et al., 2008; McLean and Fetcho, 2009; Del Bene et al., 2010; Aizenberg and Schuman, 2011; Ahrens et al., 2012). Here we demonstrate that SPIM can be used as an alternative optical method for functional imaging in zebrafish, as it also provides single- or near single-cell resolution of a very large fraction of the brain volume of larvae aged 5–9 dpf. We show that this technique further yields a ≃20-fold increase in acquisition speed (number of recorded neurons times acquisition rate) compared to point-scanning techniques without compromising the signal-to-noise ratio (SNR). We illustrate the potential of this approach by using the extended FOV provided by the SPIM technique to identify multiple brain regions exhibiting correlated spontaneous activity, and thus likely to participate in common neural processes. Since zebrafish larvae's brains are relatively small and compact (almost no extra-cellular space), we show that the *simultaneous* recording of the whole brain activity at several Hertz with single-cell resolution is within reach.

## Materials and methods

### Generation of transgenic fish

The tol2 HuC:GCaMP3 vector was built by successive ligations of a 3.2 kb fragment of the zebrafish HuC (elav3) promoter (gift from HC Park, Kyungpook National University, Korea. Park et al., 2000), then GCaMP3 calcium probe (gift from L. Looger, Howard Hughes Medical Institute, Ashburn, Virginia, USA Tian et al., 2012) into pT2KXIG in (from K. Kawakami, National Institute of Genetics, Shizuoka, Japan). HuC promoter drives the expression of a RNA-binding protein and has been involved in neuronal differentiation. In zebrafish, the 3.2 kb proximal region encompassing 2771 base pairs of the 5′-upstream sequence up to the translation start site in +383/+385, has been shown to be sufficient to target specifically and efficiently all differentiated neurons (Park et al., 2000).

One cell stage Nacre zebrafish embryos (Lister et al., 1999) (mitfa−/−) were injected with 20 ng of the plasmid DNA and 25 ng of transposase RNA (generated from pCS-TP plasmid, K. Kawakami). Injected embryos were raised to adulthood and crossed individually with Nacre fish to obtain F1 embryos. These embryos were then screened and selected according to their level of transgene expression. The embryos with the strongest expression were raised to adulthood and incrossed to obtain the homozygous HuC:GCaMP3^GS5^ line. The HuC:GCaMP3^GS5^ embryos were collected and raised at 28.5°C in E3 embryo medium. The larvae were kept under 14/10 h on/off light cycles and fed after 6 dpf. All experiments were approved by *Le Comité d'Éthique pour l'Expérimentation Animale Charles Darwin* (Ce5/2009/027).

### Larvae preparation

**Figure 1:**
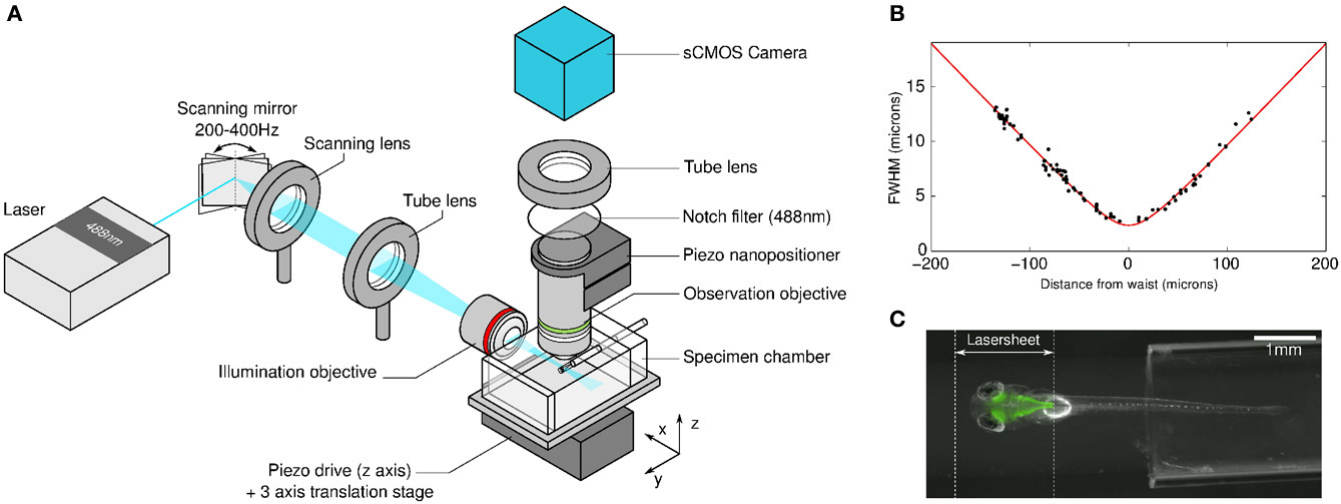
**SPIM-based functional imaging. (A)** Sketch of the optical set-up. **(B)** Laser sheet thickness profile (FWHM) along the light propagation axis. Each data point corresponds to a measurement obtained with one fluorescent bead. The red curve is the best fit of the data with the theoretical profile of a Gaussian focused beam (see Truong et al., 2011) from which the value of the waist's FWHM = 2.3 μm is deduced. **(C**) Low magnification image of the larva embedded in the agarose gel cylinder and illuminated by the light sheet.

Zebrafish larvae aged 5–9 dpf were embedded in a low-melting-temperature agarose solution at a concentration of 1.8% in embryo medium. In order to minimize movement artifacts, the solution contained 0.3 mg/ml of Pancuronium bromide, a paralyzing agent. The fish was introduced into a glass capillary tube of internal diameter 1.5 mm. The tube was then inserted inside a PMMA square chamber filled with embryo medium and the fish was partially extruded using a piece of plastic tubing inserted in the capillary tube (Figure 1C). Both sides of the specimen chamber along the illumination path consisted of glass coverslips. The larva dorsoventral axis was aligned vertically by rotation of the agarose cylinder. The chamber was then positioned in the SPIM set-up on a 3-axis manual positioning stage. A piezo-positioner (piezosystem jena PZ 400 OEM) further allowed sub-micrometric vertical displacement of the chamber.

### Optical set-up

The light sheet was obtained by rapidly scanning a focused laser beam through the specimen (Figure 1A). A 488 nm Coherent Sapphire laser beam of power 2 mW and 1/*e*^2^ diameter of 0.7 mm was projected onto a galvanometric scanning mirror (Century Sunny TSH8203) driven sinusoidally at 200–400 Hz over an angular range of 9°. A scanning lens (30 mm focal length—Thorlabs AC254-030-A), placed in front of the scanning mirror, transformed the angular deflection into a horizontal displacement of the incident light. The beam was then refocused by a tube lens (200 mm focal length - Thorlabs AC508-200-A) onto the entrance pupil of a low-NA (0.16) 5x objective lens (Zeiss EC Plan-Neofluar) facing the specimen chamber. The association of the scanning and the tube lenses extended the beam to a diameter of 4.6 mm at the entrance of the illumination objective. This optical configuration yielded a 2 mm-wide illumination sheet. Its thickness profile was characterized by imaging 100 nm in diameter fluorescent beads embedded in an agarose gel cylinder as they were scanned vertically across the laser sheet. The recorded intensity signal of each bead exhibited a Gaussian profile from which the local laser-sheet full width at half maximum (FWHM) was extracted. The sheet profile was found invariant along the scanning direction, as shown by the small dispersion obtained when plotting the measured FWHM values against the beads' distance to the waist (Figure 1B). The profile along the propagation axis was found consistent with that expected for a Gaussian beam.

The detection objective consisted of a high-NA (1.0) 20x water-immersion objective (Olympus XLUM-PLFLN) mounted vertically onto a piezo nanopositioner (piezosystem jena MIPOS 500), allowing precise adjustment of the focus plane with the light sheet. The fluorescence light was collected by a tube lens (150 mm focal length -Thorlabs AC254-150-A) and passed through a notch filter (Thorlabs NF488-15) in order to eliminate 488 nm photons. The image was then formed onto a sCMOS sensor (PCOedge). Full 16-bit images (2560× 2160 pixels) could be recorded at a maximum rate of 100 Hz directly onto RAID-0 hard drives. The 20x magnification yielded a field of view of 1 × 0.8 mm^2^, with a pixel area of 0.4 × 0.4 μm^2^. In standard experiments, images were recorded at 10–20 Hz for 20–60 min and then converted into 16-bit TIFF files.

### 3D recordings

3D recordings were obtained by sequentially imaging 5 distinct z-planes separated by a distance of 8 μm (total range of 32 μm). Every 50 ms, the specimen chamber was rapidly moved to a next position using the piezo-positioner. The sequence of the successive positions was interlaced in order to minimize the maximum step size and thus limit the acceleration imposed on the fish. The camera was asynchronously triggered 10 ms after each vertical displacement, and each frame was acquired for an exposure time of 40 ms. The 10 ms delay was necessary to allow for the agar cylinder to come to rest and prevent vibration-induced blurring of the images. At the end of the run, the frames were sorted to produce 5 separate stacks corresponding to each z-plane. Each stack was analyzed independently using the same algorithm as in single z-plane recordings.

### Automatic image segmentation

A segmentation routine written in Matlab was developed in order to automatically identify the regions of interest (ROIs) corresponding to individual somata and neuropil regions (the Matlab code is provided as a Supplementary Material). The brain contour was first manually outlined on the first image. A highly contrasted image was then obtained by time-averaging the complete images stack. Small XY drifts were corrected by registering each image of the stack with respect to the first one by extracting the displacement vector that provided the maximum correlation. The typical maximum excursion measured over an experimental run was of the order of a few microns. However small, if not corrected, this drift was found to significantly blur the time-averaged image and hamper the proper identification of individual somata. The drift displacement sequence was also used to identify periods of strong motor behaviors. As they induced significant artifacts in the fluorescence signals, the corresponding time-periods were eliminated from the analysis.

The segmentation procedure consisted of several steps. First, the image was smoothed by running a Gaussian filter with a width equal to half the typical soma diameter (5 pixels). Local contrast stretching was applied at the scale of individual neurons. A watershed algorithm was implemented on the resulting smoothed gray-scale image, returning a collection of adjacent regions associated with putative individual somata (regions of neuropil of similar area were also retained for further analysis). A few regions were then automatically eliminated based on morphological constraints (retained ROIs had a total area between 40 and 400 pixels and an equivalent ellipse eccentricity less than 0.85). The program allowed further visualization of the resulting segmentation and manual elimination of incorrectly identified neurons. Typically, a few tens of regions, essentially located at the border of the brain, were manually discarded. Given the large FOV offered by the visualization technique, up to 5,000 somata were typically imaged and their individual ROI automatically detected within a single z-plane. This segmentation procedure took approximately 1 h per 10,000 frames, with a few minutes of user time.

### Signal extraction and baseline noise estimation

The fluorescence time signal *F*(*t*) for each neuron was extracted by evaluating the mean intensity across the pixels within each ROI, in each motion-corrected image. After subtraction of the background, estimated from the average intensity of pixels outside the brain, a baseline fluorescence signal was estimated for each neuron by the running average of the 8th percentile of the raw data in sliding windows of 30 s length (Dombeck et al., 2007). The resulting smooth curve *b*_slow_ locally approximated the baseline level and reflected slow fluctuations unrelated to the fast calcium transients evoked by spiking activity. The relative variation of fluorescence intensity, *dF/F*, was calculated as *dF/F* = (*F(t)* − *b*_slow_)/*b*_slow_.

The standard deviation σ_noise_ of each neuron's baseline fluctuations was extracted from the distribution of neuronal *dF/F* values. These distributions are skewed toward positive values reflecting the presence of activity-evoked positive transients in the fluorescence signal. In contrast, negative data points are unlikely to be related to neuronal firing. The standard deviation of the baseline noise was thus estimated by postulating that these negative fluctuations were part of a Gaussian stochastic process. Consistently, the corresponding region of the distribution could be accurately fitted with a Gaussian function (*r*^2^ = 0.997 ± 0.002 for a representative dataset) from which the standard deviation σ_noise_ was extracted for each neuron's fluorescence time-series.

### 2P-PSM experiments

In order to provide a comparison between SPIM and 2P-PSM approaches, neural recordings of spontaneous activity in the tectum were performed on similar GCaMP3-expressing larvae using a MOM-sutter system. Its main components consisted of a 25x NA1.05 Olympus objective and a Mai Tai DeepSee Ti:sapphire laser used at 920 nm with an output power of less than 3 mW after the objective. The filters consisted of a FF705 dichroic, a AFF01-680 short path (IR Blocker) and a FF01520/70 band-pass filter, all from Semrock. The PMT was a H1070 (GaAsP) from Hamamatsu. Images of 256 x 256 pixels were acquired at 4 Hz.

### Analysis of neuronal activity correlations

For this analysis, we only considered fluorescence transients inferred to result from neuronal firing by imposing a threshold on each individual time-series. Any *dF/F* value below 3 σ_noise_ was set to 0. For each dataset, 100 independent runs of the K-means algorithm with squared Euclidean distance and random seeds were performed (MacQueen, 1967). Only the run with minimal total sum of distances was retained. In order to determine the total number *K* of clusters in the segmentation, a sweep in *K* space between 1 and 20 was performed, and the results were inspected with silhouette plots. For example, when analyzing the data displayed in Figure 5, *K* = 12 was found to be the best solution. Importantly, the neuronal clusters shown in this figure, which are the most compact, proved robust with respect to changes in *K* between 10 and 20.

## Results

Our main objective was to establish the advantages and limitations of SPIM for *in-vivo* functional imaging in zebrafish. Spontaneous activity was recorded in 5–9-day-old HuC:GCaMP3 zebrafish larvae. First, we characterized the volume of the brain accessible to single-cell calcium imaging using SPIM. Second, we estimated the gain in the number of neurons that could be simultaneously sampled, and in the maximum accessible acquisition rate, that this imaging strategy provides compared to standard 2P-PSM experiments. Finally, we showed how the extended FOV provided by SPIM allows one to probe correlations in spontaneous activity among multiple brain regions as an indication of inter-regional brain connectivity.

### Optical sectioning efficiency of the spim set-up

In SPIM, the efficiency of the optical sectioning is set by the thickness of the illumination sheet. As expected for scanned illumination with a Gaussian beam, the laser sheet profile was found to be invariant along the scanning axis and to display a hyperbolic profile along the light propagation axis, with a diffraction-limited minimum thickness of 2.3 μm located at the focal plane of the illumination objective (see Figure 1B). This value was found to increase to 7 μm at a distance of 80 μm from the waist. Given the characteristic inter-neuron distance (of the order of 7 μm), the method thus yields single neuron resolution over a FOV of ≃160 × 1000 μm around the midline of the larva, a region which contains the majority of the somata. In the most distal regions of the zebrafish brain, the illumination sheet spans 1–2 neuron diameter such that SPIM does not provide single-cell resolution.

**Figure 2:**
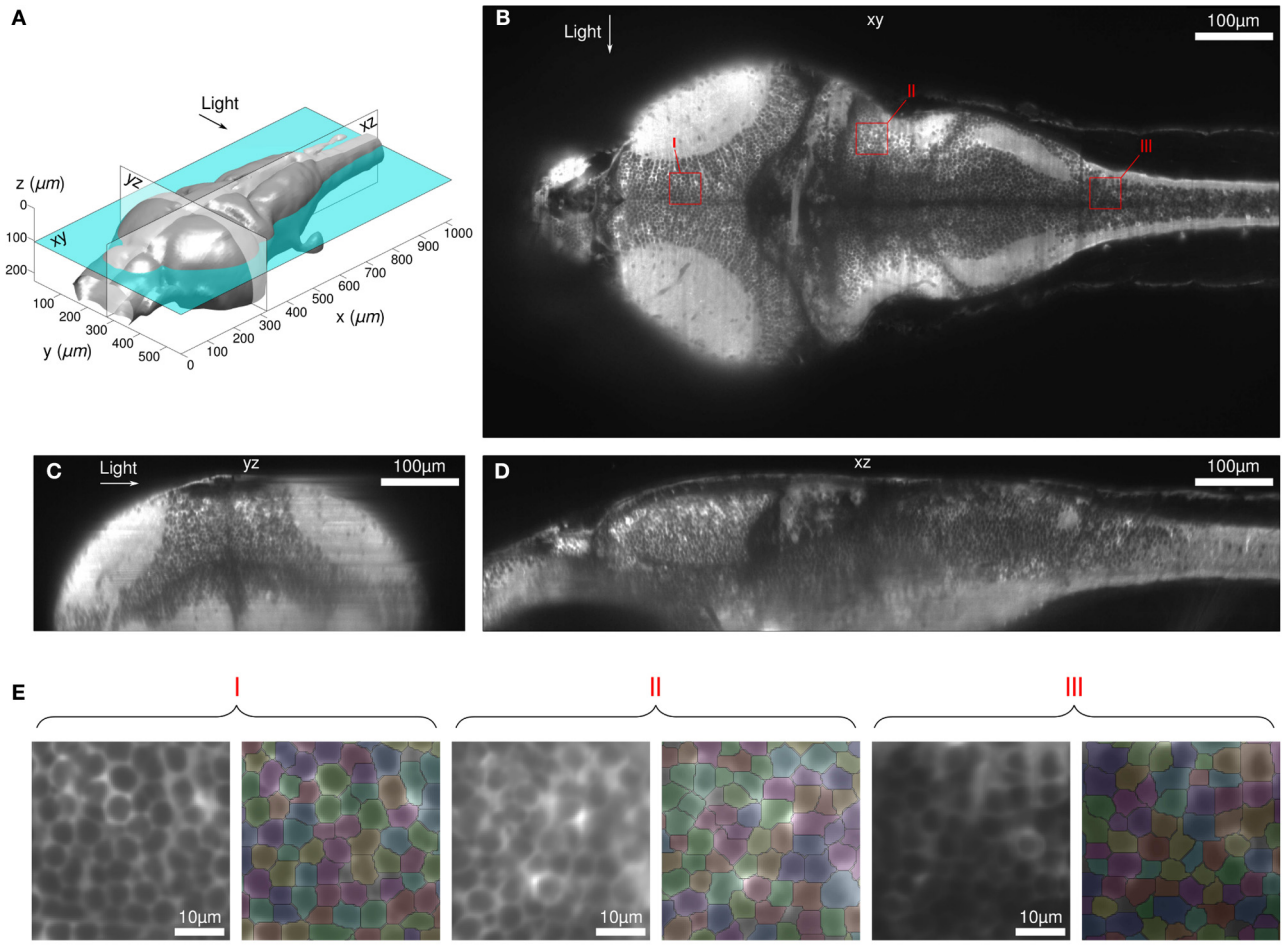
**Stack and segmentation. (A–D)** Multi-view sections of the brain-volume reconstructed from a complete 3D stack (no filtering). The voxel volume is 0.4× 0.4 × 0.4 μm^3^. See Supplementary Movie 1 for a complete visualization of the brain. **(E)** Result of the segmentation algorithm for 3 different sub-regions shown in **(B)** with red-squares.

Despite this limited axial resolution, the images appeared highly contrasted in most of the brain volume, allowing for the implementation of an automatic segmentation algorithm in order to identify ROIs associated with each neuron. This aspect of the method is illustrated in Figure 2. Images were taken at successive z-positions with 0.4 μm intervals across the brain over a total vertical distance of 220 μm (see Supplementary Movie 1). The exposure time was set to 100 ms such that the complete stack was acquired in less than 1 min. We were able to automatically identify virtually all the somata throughout the brain volume except for small telencephalic regions located in the shadow of the eye and in the neuropil regions.

The robustness of the automatic segmentation procedure is illustrated in Figure 2B for different brain areas at a depth of 104 μm, which corresponded to the middle of the preceding stack. The segmentation was performed on the image obtained by time-averaging 18,000 frames acquired at 10 Hz over 30 min, which corresponds to a typical neural recording configuration. The possibility to automatically identify somata's ROIs is a crucial aspect of the present approach, since the manual outlining of ≃5,000 neurons per plane would otherwise impose a severe practical limit on the method.

One should be aware that the light sheet thickness measurement described in Materials and Methods provides an upper bound for the axial resolution in the actual experiment. When imaging the brain volume, the scattering of the illumination beam tends to reduce the effective axial resolution. Similarly, the x-y resolution is expected to be degraded by the scattering of the fluorescence photons. The magnitude of these effects was estimated by computing the cellular-scale contrast, defined as the difference in intensity between the brightest and dimmest pixels (second and 98th percentile) over areas slightly larger than the somata characteristic ROIs. As anticipated, the contrast was found to be maximum in the most dorsal region of the fish, where the distances traveled by the illumination and fluorescent photons were both minimum. The contrast then decreased continuously with the depth of observation, but with a moderate decay rate of 40± 5% per 100 μm.

### Fast, brain-wide calcium imaging

**Figure 3:**
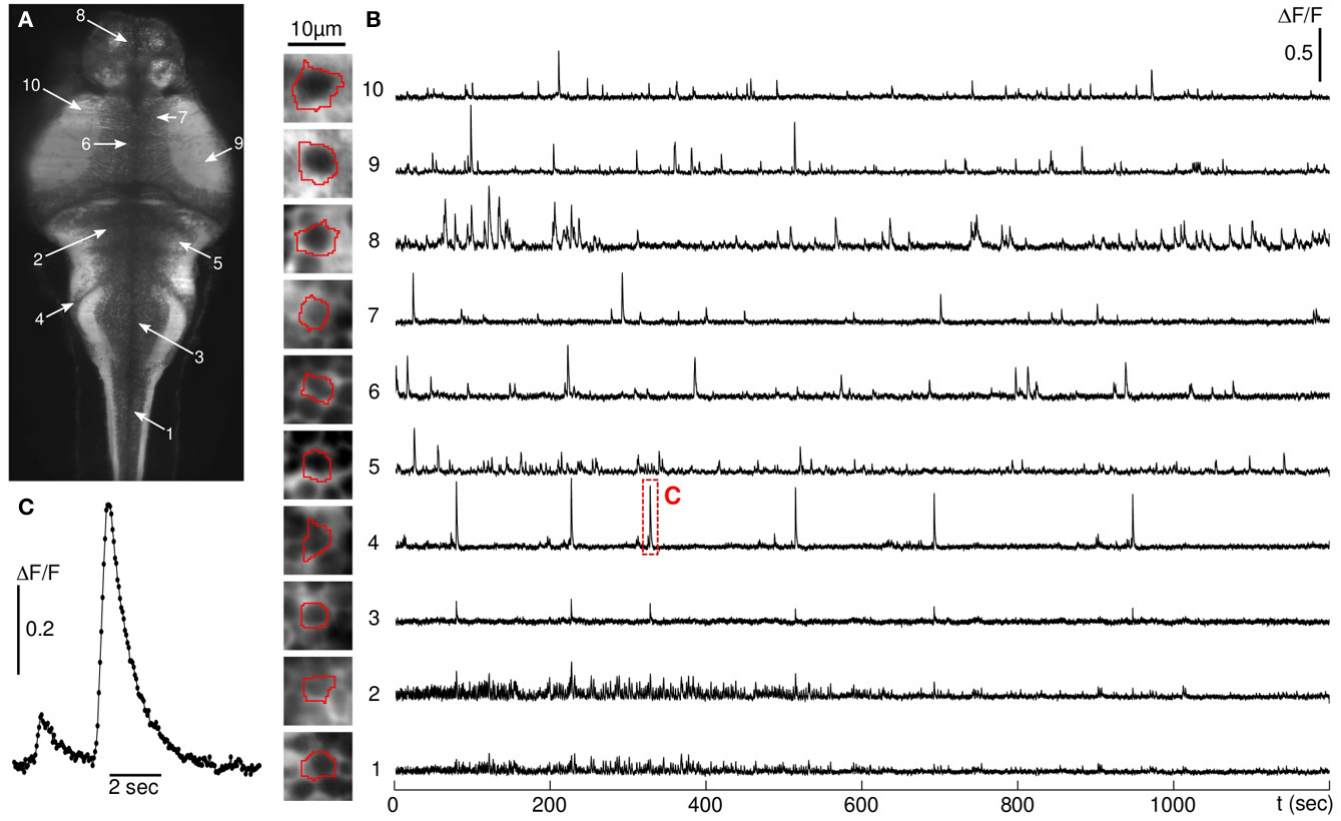
**Fluorescence signals. (A)** Time-averaged image of a brain slice of a 6 dpf larva. **(B)** Individual traces of 10 neurons whose locations are indicated in red on the image, recorded at 20 Hz acquisition rate. the thumbnails display each neuron's ROI. **(C)** Blow-up view of a typical activity-related fluorescence event indicated by a red rectangle in **(B)**.

Images of a single z-plane were acquired at 10–20 Hz (50–100 ms exposure time) over periods of 20–60 min (a short excerpt of a 10 Hz recording is provided in supplementary Movie 2). In the majority of the experiments, the plane of observation was chosen to encompass a large number of neurons from all major brain regions (spinal cord, hindbrain, midbrain, and forebrain). After automatic segmentation, the relative fluorescence time series *dF/F* was extracted for each individual soma and for neuropil regions of similar area (Figure 3).

As no external stimulation was applied, the recorded fluorescence dynamics reflected spontaneous neural activity. Strictly speaking, the illumination wavelength (488 nm) being within the fish visual spectrum, the larva was submitted to a visual stimulation. However, owing to the rapid scanning rate (>200 Hz), such a stimulation can be considered physiologically time-invariant and the fish is expected to habituate rapidly. Consistently, we did not observe any systematic change in the level of neural activity over the duration of the experiments. This was checked by computing the mean of the activity-related transients in the first and second half of the runs. The difference between both measurements was not found to be statistically significant.

In all runs, the laser intensity was set to 2 mW. At this power, the fluorescence baseline did not display any noticeable decay over the duration of the experimental runs, which indicated the absence of significant photo-bleaching. Furthermore, as already mentioned, the level of activity showed no systematic change. Both observations provided a solid indication that phototoxicity effects should be rather limited at this level of illumination. At 6 mW, although the level of photo-bleaching was still relatively modest (≈10% baseline intensity decay in 20 min), the activity-related signal from somata in all regions of the brain tended to vanish. This effect was reversible, i.e. the original fluorescence transient level could be recovered by setting the power back to 2 mW. The most plausible interpretation of this set of observations is that before phototoxicity effects become significant, the calcium-bound fluorophores, which have a much higher quantum yield that the unbound species, reached their photo-emission saturation rate resulting in a significant reduction in amplitude of the fluorescence transients.

In some experiments, streaking artifacts were observed in the form of rapid correlated fluctuations of intensity along narrow bands oriented parallel to the illumination axis. These flickering regions resulted from the motion of light absorbing objects (probably the hemoglobin within blood cells) circulating along the exposed face of the animal. As they crossed the light sheet, they projected a thin shadow along the illumination path. When too intense, these fluctuations biased the analysis of the fluorescence signals, so that the corresponding neurons had to be manually eliminated from the analysis.

### Three-dimensional calcium-imaging

**Figure 4:**
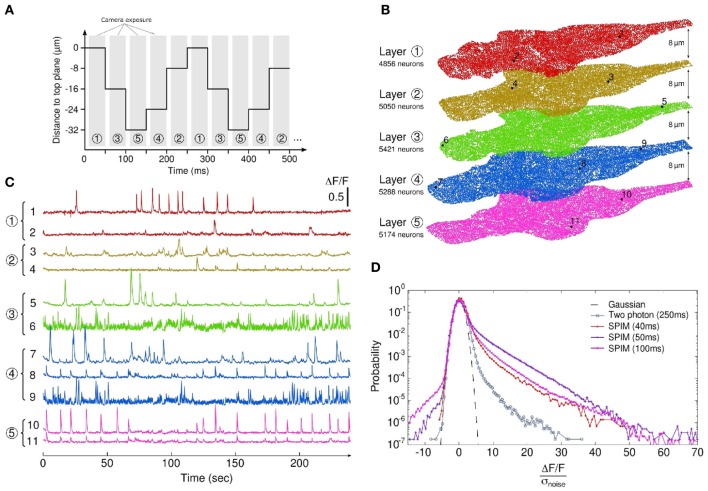
**3D calcium-imaging. (A)** Interlaced sequence of acquisition of 5 z-planes separated by 8 μm intervals. Exposure time is 40 ms and each plane is recorded at a rate of 4 Hz. **(B)** Localization of the monitored ROIs (somas and neuropil regions) obtained through automatic segmentation on the 5 different planes. The number of actual somas is given for each z-plane, yielding a total of 25789 simultaneously recorded neurons (see Supplementary Movie 3 for the 5 z-frames fluorescence dynamics). **(C)** Characteristic signals from neurons within the stack. **(D)** Comparison of the noise-normalized fluorescence signal distributions for SPIM-based experiments (colored) and a 2P-PSM experiment (gray). For each experiment, the exposure time is indicated. The 40 ms-exposure SPIM experiment is the 3D imaging run (5 z-planes, 4 Hz effective recording rate).

The high data throughput provided by SPIM can be used to obtain dynamic 3D brain-wide recordings of neural activity at a lower acquisition rate. As a proof of concept, we recorded 5 different z-planes, separated by 8 μm, a distance which guaranteed that neurons from different slices were distinct. Axial scanning was obtained by sequentially moving the specimen at the successive z-positions (Figure 4A). This approach allowed for the simultaneous recording of over 25,000 individual neurons (and large neuropil regions) at 4 Hz. Figures 4B,C shows the location of the centers of mass of the sampled ROIs and the associated *dF/F* traces for a few neurons, located in different regions of the brain.

### Evaluating the signal-to-noise ratio (SNR)

In order to evaluate the sensitivity of the method, a statistical comparison was made between spontaneous activity recordings obtained using SPIM and 2P-PSM in the tectal region under comparable experimental conditions (see Materials and Methods, all statistics were computed from three independent runs for each set-up). At 10 Hz acquisition rate, the standard deviation σ_noise_ of the baseline fluorescence signal (extracted for each neuron, see Materials and Methods) using SPIM was found to be of order 1% (σ_noise_ = 0.008 ± 0.002). This quantity was measured at 0.13±0.04 in 2P-PSM at 4 Hz acquisition rate. The relatively low noise level observed in SPIM is a consequence of the larger photon count provided by the method. A significant fraction of these photons, however, originates from regions outside the in-focus ROIs, owing to the less efficient sectioning and the wide-field nature of this optical method. This in turn increases the background signal, thus limiting the amplitude of the *dF/F* transients. For comparable configurations, 2P-PSM recordings actually displayed statistically higher *dF/F* peak signals: the average of the *dF/F* values within the highest percentile was found to be 0.11 and 0.55 in SPIM and 2P-PSM, respectively.

To provide a fair SNR comparison between both methods, each neuronal *dF/F* distribution was thus normalized by its baseline noise standard deviation σ_noise_. The resulting noise-normalized fluorescence distributions are shown in Figure 4D for three experimental SPIM runs (single z-plane recordings at acquisition rates 10 and 20 Hz, 5 z-planes 3D recording at 4 Hz) and for a typical run performed at 4 Hz using 2P-PSM. Although the SPIM experiments used lower exposure times (40–100 ms instead of 250 ms), the corresponding distributions appeared to be more significantly skewed toward large positive values as compared to 2P-PSM, which indicated that the SNR was improved. Across all experiments, the skewness of the noise-normalized distribution was found to be 3.70±0.78 in SPIM as compared to 0.54±0.11 in 2P-PSM. Although this difference is statistically significant, one should consider such a determination of the relative SNR as a rough estimate since the level of spontaneous activity in the tectum may vary significantly between runs.

### Identifying brain-wide highly correlated neuronal clusters

One of the important assets of the SPIM method, with respect to PSM approaches, lies in the possibility of simultaneously recording neural activity in distinct brain regions. This in turn enables the identification of ensembles of neurons spanning different regions of the brain that exhibit correlated activity and are thus likely to be interconnected or activated by a common source. Here we illustrate this possibility by using a 60 min long single plane recording of multiple brain regions at 10 Hz acquisition rate. The fluorescence time-series of each neuron was first thresholded in order to extract activity-related events (see Materials and Methods). The rest of the data points was set to zero. This thresholding was necessary to eliminate coherent baseline noise, induced by minute laser intensity fluctuations, which would have otherwise biased the correlation analysis.

**Figure 5:**
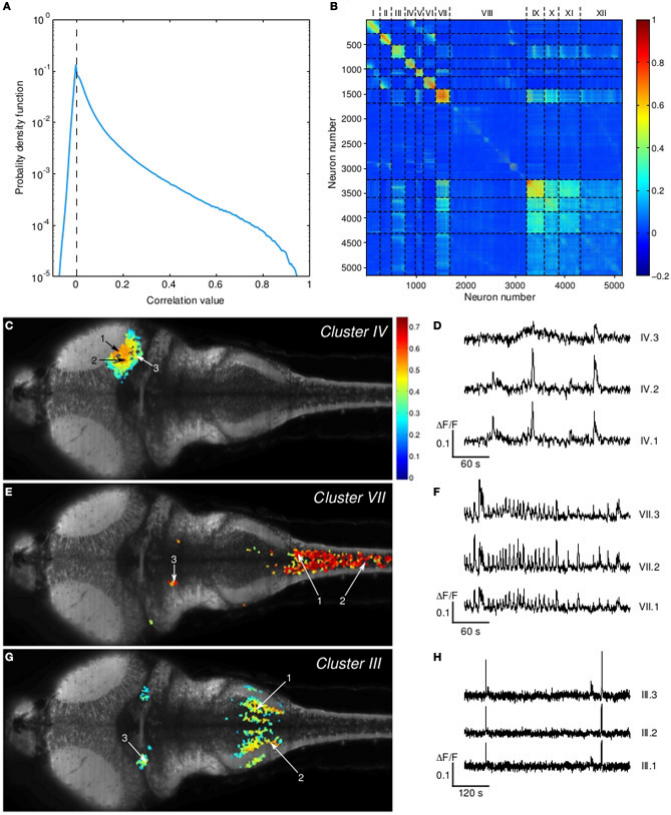
**Activity-correlated neuronal clusters. (A)** Distribution of pair-wise correlations. The data were obtained from a 10 Hz, 60 min long experiment on a 6 dpf larva. **(B)** Correlation matrix sorted using the K-means algorithm with associated cluster partitioning. **(C–G)** Topographical layouts of clusters IV-VII-III. The color code corresponds to the mean pair-wise correlation value with the neurons from the cluster. For each cluster, 3 short excerpts of fluorescence traces exhibiting clear activity correlation are shown **(D–H)**.

The distribution of pair-wise correlations showed a heavy-tail of fairly large values, suggesting the presence of significantly correlated activity (Figure 5A). As a first attempt to identify the optimal clustering of the neural activity time-series, the K-means algorithm was implemented (see Materials and Methods). An example of the computed partitionings is shown in Figure 5B, where a reasonable clusterization of highly correlated neuronal groups with moderate cross-talk can be observed. Three of these clusters topographical layouts are shown in Figures 5C–G, together with excerpts of (unfiltered) fluorescence traces of 3 neurons within each cluster.

The first cluster revealed significant correlations among neighboring neurons in the optic tectum (Figure 5C). Notice that the mean pair-wise correlation was found to continuously decay from a confined region located at the border of the neuropil. The second cluster consisted of neurons mostly located in the spinal cord (Figure 5E). In the first 10 min of the run, these neurons exhibited characteristic rhythmic synchronous activity at a typical frequency of 0.2 Hz (see Figure 5F). In the paralyzed preparation, these bouts of spinal cord activity are likely to be associated with fictive swimming events. The large FOV allowed us to further identify a sub-population of distant neurons located in two bilaterally symmetric confined regions, which probably correspond to the medial octavolateralis nucleus. A similar cluster of correlated neurons was observed in another animal exhibiting fictive swimming. Finally, stripes of neurons located in the vagal lobe, a brain structure implicated in the processing of gustatory stimuli, were found to be correlated with distant bilaterally symmetric neuronal clusters probably located at the secondary gustatory nuclei (SGN), in the isthmus ventrally to the cerebellum (Figure 5G). It should be noticed that these three functional clusters, associated with three distinct modalities (visual, motor, and gustatory), were identified from a single 60 min long recording of a single z-plane.

## Discussion

In PSM-based calcium-imaging, neurons are probed sequentially, which imposes a trade-off between the number of sampled neurons *N*_cells_ and the acquisition rate *f*_acq_ (Lütcke and Helmchen, 2011). In order to increase the maximum of the product *N*_cells_*f*_acq_, AOM-based random scanning approaches have been developed, which aimed at minimizing the time wasted in acquiring regions outside the neurons volume. Notice that this issue is almost irrelevant in zebrafish, since most of the brain consists of tightly packed somata (almost no extra-cellular space). A fundamental limit in data throughput is, however, set by the minimum dwelling time of the laser at each voxel required to record a significant number of photons.

Scanned laser sheet imaging also relies on sequential recording of neurons, but those within a given line are illuminated and recorded in parallel. As established in the present experiment, this yields a ≃ 20−fold increase in the accessible *N*_cells_*f*_acq_ product for similar experimental conditions. Hence, while current PSM-based calcium-imaging set-ups in zebrafish allow monitoring ≃1000 neurons at 5 Hz, SPIM allows monitoring up to 5,000 neurons from a single z-plane at 20 Hz, and over 25,000 neurons from 5 different z-planes at 4 Hz, with similar or better SNR. This large increase in data throughput offered by SPIM-based calcium imaging opens new opportunities for studying various aspects of neural processes.

### Toward *in toto* dynamic neural recording of a vertebrate brain

Larval zebrafish constitutes a unique vertebrate system to study the activity of brain-wide neural circuits and help decipher the way information is processed across different brain regions. This possibility was illustrated in a recent paper by Ahrens et al. (2012). By recording calcium activity in an animal that interacted fictively with a virtual environment, the authors were able to identify neural populations spanning multiple brain areas that were activated during specific phases of adaptive locomotion. Owing to the limited number of neurons sampled during a single recording, different experiments and animals had to be fused in order to produce a complete physiological map of the associated neural networks. This approach was not only time-consuming but also implicitly assumed that the recruited neuronal population was invariant from run to run and animal to animal. In order to overcome these limitations, one would need to *simultaneously* record the complete brain. This would allow for the gathering of similar data on a single-trial basis, and thus permit one to probe the variability in the spatial pattern of the recruited neural population.

In its current configuration, our SPIM set-up allows one to simultaneously sample ≃30% of the total number of neurons (Hill et al., 2003) at 4 Hz. Given the decay time of GCaMP3 (of the order of 0.5 s), this acquisition rate guarantees that any calcium transient for a given neuron within this volume is detected. This result was shown as a proof-of-concept and further refinements are needed. In particular, axial scanning was obtained by continuously moving the sample across the light-sheet. This approach presents two major drawbacks. First, it submits the larva to an oscillatory acceleration of maximum amplitude of order 0.1 ms^−2^ = 10^−2^ g where *g* is the gravitational acceleration. Although relatively low, this inertial stimulation may trigger responses from the fish's vestibular system. Second, the softness of the agar gel limits the rate at which successive z-positions can be imaged, since a significant time is needed for the cylinder to come to rest. Both issues could be addressed by maintaining the specimen fixed while synchronously moving the laser sheet (using a second galvanometric mirror) and the observation objective. Higher z-scanning rates could also be obtained by using tunable optical components (Grewe et al., 2011). Given the constant progress in GECI sensitivity (Akerboom et al., 2012) and sCMOS sensors, SPIM-based calcium imaging should provide virtually full brain simultaneous recording at several Hertz in the near future.

### Probing the time-structure of brain-wide neural activity patterns

Calcium-imaging was originally seen as a way to overcome the limitation in the number of neurons that could be recorded using electrophysiological approaches. For a long time, it was believed that an inevitable corollary of this method was to sacrifice temporal information as the calcium signals were typically probed at a few Hertz, far below physiological timescales. In recent years, several fast imaging experiments proved this assumption wrong. Recently, Grewe *et al.* demonstrated a ≃10 ms resolution on the firing dynamics of individual neurons based on calcium-imaging performed at hundreds of Hertz (Grewe et al., 2010). This time-resolution was actually set by the intrinsic dynamics of the fluorescent calcium indicator (Oregon Green BAPTA-1). This achievement demonstrated that calcium-imaging could constitute a suitable method for probing neural networks dynamics with near-millisecond resolution, and thus could serve to explore how spike-timing may play a role in neural processes. However, this exciting result was obtained at the cost of the number of probed neurons, of the order of a few tens *i.e.* close to that currently recorded using silicon-based nanoprobes. Fast *in vivo* neural recordings are thus currently restricted to spatially confined micro-circuits. In contrast, SPIM should allow reaching high acquisition rates while still probing a large number of neurons. In the present experiments, the acquisition rate was limited to 20 Hz since the relatively slow GCaMP3 rising time (of the order of 100 ms) sets the bandwidth within which spiking dynamics can be efficiently probed. Increasing the acquisition rate would only degrade SNR without enhancing the time-resolution. With sufficiently rapid and sensitive calcium reporters—such as the synthetic sensors used by Grewe et al. (2010)—it should be possible to record up to 1000 neurons with near-millisecond resolution.

Several limitations of this method still need to be addressed. First, the optical sectioning only guarantees single-cell resolution in the most proximal part of the brain. Different methods could be implemented to increase the axial resolution, such as structured illumination (Keller et al., 2010) or confocal light-sheet imaging (Silvestri et al., 2012). A second issue is related to the use of visible light illumination (488 nm), which currently hampers the proper stimulation of the fish visual system. This limitation could be circumvented by using two photon fluorescence excitation with the same SPIM geometry (Palero et al., 2010; Truong et al., 2011). This would in turn opens the possibility to implement Bessel-beam illumination. Beyond the expected gain in optical sectioning efficiency provided by this method (Planchon et al., 2011), the self-healing property of Bessel beams may permit to mitigate the problems of streaking.

In the present article, we have demonstrated how the extended FOV provided by this alternative imaging strategy allows one to identify brain-wide functional neuronal circuits by straightforward correlation analysis of the spontaneous activity pattern. Overall, the combination of SPIM-based functional imaging, transgenic larvae and methods for statistical data analysis will lead to the generation of a whole brain atlas of functional neuronal connectivity, either by monitoring spontaneous or sensory-evoked activity, or through localized channelrhodopsin-2 stimulation.

### Conflict of interest statement

The authors declare that the research was conducted in the absence of any commercial or financial relationships that could be construed as a potential conflict of interest.
